# Gender Dysphoria: The Role of Sex Reassignment Surgery

**DOI:** 10.1155/2014/645109

**Published:** 2014-07-10

**Authors:** Miroslav L. Djordjevic, Christopher J. Salgado, Marta Bizic, Franklin Emmanuel Kuehhas

**Affiliations:** ^1^School of Medicine, University of Belgrade, Serbia; ^2^School of Medicine, University of Miami, FL, USA; ^3^University of Vienna, Austria

This special issue represents a comprehensive, multidisciplinary look on gender dysphoria. We invited investigators to contribute original research articles as well as review articles that will further encourage the continuing efforts to understand the role of gender dysphoria, sex reassignment surgery, modalities of surgical treatment, and the patient's evaluation of hormonal, psychosocial, and psychosexual outcomes. We were particularly interested in articles describing new operative procedures, the modifications of old ones, the modalities of postoperative follow-up, the role of hormonal support, and the recommendations for the future. As a result, we have assembled this special issue with eight excellent papers providing authoritative and concise information on gender dysphoria patients and covering almost all aspects of their treatment.

In “*An overview of neovaginal reconstruction options in male to female transsexuals,”* M. Bizic et al. reviewed numerous methods that have been described for vaginal reconstruction in the treatment of transgenders. The choice of surgical option for vaginoplasty depends on the surgeon's experience as well as on the patient's wishes and expectations. Sometimes a multidisciplinary approach is necessary in prevention of postoperative complications and poor psychosocial and psychosexual outcome. S. Vujović et al. investigated the importance of prenatal hormone exposure in the development of transsexualism by measurement of finger lengths in a sample of Serbian patients. In their paper entitled “*Finger length ratios in Serbian transsexuals,”* both FtM and MtF patients were included and compared to cisgendered females and males. The results obtained support the biological origins of transsexualism.

The authors of “*Personality disorders in persons with gender identity disorder”* assessed personalities and personality disorders (PD) by comparing gender dysphoric and cisgender persons using the art's most frequently applied instrument, Structured Clinical Interview (SCID-II). Duisin et al. obtained significant results, some of which were similar to previous studies, while others diverged. Evaluation of PD's comorbidity with gender dysphoria is important for the optimization of treatment during the pretransitory and transitory periods, but for some patients it is relevant in the postoperative period as well. A paper entitled “*Assessment of self-perception of transsexual persons: pilot study of 15 patients”* by J. Barišić et al. investigates the self-perception of transsexuals by using the Rorschach test, an important and useful tool in psychological examination of candidates for sex reassignment surgery. The value of this pilot study lies in its examination of adaptive capacities for transition, which is of great importance in prediction of the outcome.

The complex anatomy of the clitoris is a subject area with which reconstructive surgeons must remain completely abreast. In their paper “*The role of clitoral anatomy in female to male sex reassignment surgery*,” V. Vukadinovic et al. confirmed the significance of comprehensive understanding of the clitoral anatomy as a basis for a successful outcome after metoidioplasty. In addition, they pointed out the role of the clitoris in achieving a normal sexual life after transition from female to male, following either metoidioplasty or total phalloplasty. Preliminary results from an Italian group of researchers, described by S. Bogliolo et al. in “*Robotic single-site surgery for female-to-male transsexuals: preliminary experience,”* showed us the importance of minimally invasive principles for hysterectomy and bilateral oophorectomy in FTM patients. Following the experience of laparoscopic approaches in reconstructive surgery, authors take a step beyond with recommendations on robotic surgery.

The paper entitled “*Psychosocial adjustment to sex reassignment surgery: a qualitative examination and personal experiences of six transsexual persons in Croatia”* presents N. Jokić-Begić's personal clinical experience in the specific cultural and religious environment of the Balkans. Exchange of the clinical observations of insights from the personal lives of transgender persons worldwide is significant, as it creates a medical and scientific ambience for overcoming the difficulties and obstacles gender dysphoric patients face throughout the process of transition and psychosocial adjustment after SRS. The authors stress the importance of social and medical support which sometimes highly correlates with the prolongation of transition period.

Finally, the paper from P. Tiewtranon'sgroup entitled “*The development of sex reassignment surgery in Thailand: a social perspective*” retrospectively evaluated the development of surgical procedures in Thailand. It describes how rich experience teamed with a dedication to surgical excellence provides the ideal environment for successful surgical treatment of transsexuals. The experiences described prove that guidelines for sex reassignment surgery are both simple to understand and useful to apply.

